# “I'm Dr Jekyll *and* Mr Hyde”: Are GPs’ antibiotic prescribing patterns contextually dependent? A qualitative focus group study

**DOI:** 10.3109/02813432.2013.824156

**Published:** 2013-09

**Authors:** Eva Lena Strandberg, Annika Brorsson, Charlotta Hagstam, Margareta Troein, Katarina Hedin

**Affiliations:** ^1^Lund University, Department of Clinical Sciences, Family Medicine, Malmö, Sweden; ^2^Blekinge Centre of Competence, Blekinge County Council, Karlskrona, Sweden; ^3^Primary Health Care, Region Skåne, Malmö, Sweden; ^4^Unit for Research and Development, Kronoberg County Council, Växjö, Sweden

**Keywords:** Antibiotic prescribing, consultation, doctor–patient relationship, general practice, primary health care, respiratory tract infections, Sweden

## Abstract

**Objective:**

To explore factors and circumstances contributing to prudent antibiotic prescribing for respiratory tract infections in primary care.

**Design:**

Two focus groups representing rural and urban areas. A semi-structured interview guide with open-ended questions and an editing analysis style was used. They were examined to identify meaning units that were sorted into categories in an iterative process throughout the analysis.

**Setting:**

Primary health care in two counties in southern Sweden.

**Subjects:**

Two groups including seven and six general practitioners (GPs) respectively, men and women of different ages with different professional experiences.

**Main outcome measures:**

Exploration of categories, determination of themes, construction of models.

**Results:**

The decision to prescribe antibiotics takes place in the encounter between GP and patient, initially characterized by *harmony* or *fight* and the subsequent process by *collaboration* or *negotiation*, resulting in *agreement, compromise*, or *disagreement*. Several factors influence the meeting and contribute to enhancing the conditions for rational prescribing. These conditions are connected to the GP, the relationship, and the setting; organization as well as professional culture. The findings indicate synergies between the factors, and that one factor can sometimes compensate for lack of another. Continuity and mutual trust can make a brief consultation successful, but lack of continuity can eliminate the effects of knowledge and professional skills.

**Conclusions:**

The findings emphasize the importance of the encounter between the GP and the patient for prudent antibiotic prescribing. Furthermore, the importance of an appropriate organization of primary care, which promotes continuity and encourages professional autonomy, is demonstrated.

In spite of numerous efforts to reduce unnecessary antibiotic prescribing, the problem remains. This study emphasizes:

- The importance of the doctor–patient encounter for prudent antibiotic prescribing.
- An appropriate primary care organization that promotes continuity and professional autonomy.

## Introduction

Respiratory tract infection (RTI) is still one of the most common reasons for encounters in primary health care. Most respiratory infections are viral, and many of the bacterial infections are self-limiting, but antibiotics are frequently prescribed [1]. This over-use has increased antibiotic resistance, and there is evidence that resistance develops not only on the aggregated level but also in the individual [2].

The reasons for prescribing antibiotics contrary to current guidelines have been discussed and studied extensively [3–6]. In a Canadian historical cohort it was shown that inappropriate antibiotic prescribing increased with time in practice and was also more frequent among foreign medical graduates and among primary care physicians with a high practice volume [3].

A well-known problem is the constant prescribing pattern on an individual basis. Cars and Håkansson concluded that “Doctors have an individual and very constant pattern of prescribing antibiotics, and it seems that the diagnoses are often given to justify the treatment, rather than the other way around” [4]. Some other studies concluded that a high frequency of prescriptions of antibiotics may reflect a general disposition among GPs to give priority to maintaining good relations with the patients [5,6]. They suggest that low antibiotic prescribing and high patient satisfaction could be combined if sufficient time were spent on listening to patients. Patient satisfaction was not related to the prescription of antibiotics, but to a better understanding of their illness.

Numerous efforts have been made to reduce unnecessary and potentially harmful antibiotic prescribing. These include education for providers, near-patient tests, and the introduction of financial incentives, as well as information to patients and public [7–10]. Case-based and interactive education has been proved to be effective in improving prescribing policies [2,7]. Attitudes were considerably changed, and this corresponded with altered prescribing patterns [2]. A Cochrane Review from 2005 advocated combined education for physicians, patients, and the public [11]. In a Swedish follow-up study after education, an overall reduction in the prescription of antibiotics was seen, and the results were persistent several years later [12].

A recent study has revealed that great differences in prescribing habits remain between different countries, geographical areas, and practices [13,14]. In order to find paths to further improvement of prescribing habits, we considered it essential to deepen the knowledge concerning facilitating factors and barriers to rational prescribing.

Accordingly, the aim of this study was to explore general practitioners’ (GPs) perceptions and experiences regarding antibiotic prescribing for RTIs in Swedish primary care.

## Material and methods

### The APO method

This paper reports on one component of the EU project HAPPY AUDIT [13].The Audit Project Odense (APO) method was tested to change antibiotic prescribing habits in six countries, chosen for their differing prevalence of antibiotic resistance. The audit was performed twice, with a year's interval during which an intervention took place. Considerable changes in prescribing habits were registered [13,14]. However, in Sweden, substantial differences between practices remained after the intervention. This suggests that the application of guidelines varies due to factors other than medical.

### Study design

Focus groups were used to explore GPs’ perceptions of antibiotic prescribing for RTIs in primary care. Focus groups are effective for exploring the meaning of conceptions and definitions of important phenomena in health care [15,16].

The interactive discussions of focus groups generate valuable details of complex experiences and reasons behind actions, beliefs, perceptions, and attitudes [17,18]. Focus-group interaction facilitates in-depth discussions through a sequence of open-ended questions that encourage participation within the group. Group dynamics create a synergy between participants contributing to discussions and descriptions of individual experiences, underlying opinions, meanings, feelings, and attitudes. A central element is not only the content of the discussion, but also the interaction between participants [19].

All authors have research experience. AB, ELS, and MT have done qualitative research using focus groups. ELS has worked with quality development and process supervision in various primary care audit projects. The other authors are general practitioners with experiences from prescribing antibiotics for RTIs.

### Data collection

Two focus groups with six and seven participants from the Happy Audit project were arranged in September 2009 in two counties in the south of Sweden (Table I). The participants were both men and women; GPs of different ages and professional experience, and from both rural and urban areas participated. Each group met once for a 60-minute session. We used a semi-structured interview guide with open-ended questions [20]. Topics for the focus group discussions were (i) Experiences of antibiotic prescribing for RTIs and personal behaviour, (ii) Experiences of deviating from national and local guidelines, (iii) The impact of educational interventions such as the Happy Audit project.

**Table I.** Distribution of participating GPs.

Category	Variables	n
Gender	Women	10
	Men	3
Age	18–39 > 40	2 11
Practice type	Group	13
Location	City	6
	Town	1
	Village	6

A moderator introduced each focus, and made sure that participants did not deviate from the research question and that everyone was able to take part in the discussion. An assistant took notes, which were added to the data from the audiotaped discussion that was transcribed verbatim. To validate the focus-group data, ELS and AB did three individual telephone interviews with Happy Audit participants from a third Swedish region.

### Data analysis

An editing analysis style according to Miller and Crabtree [21] was used starting with a naive reading followed by repeated readings of all transcripts that were examined independently in order to identify meaning units or segments that both stand on their own and relate to the aim of the study, but separate from preconceptions prior to reading the data. The units or segments were sorted and organized into categories in an iterative process throughout the analysis. In the further analysis the authors explored categories, determined patterns and themes, and constructed models. The continued analysis included exemplar quotes illustrating each area (Table II). ELS and AB met on 10 occasions identifying similarities and connections and discussed until consensus was reached. Preliminary findings were presented to the participants for feedback. CH, MT, and KH read the focus-group interviews and confirmed that they contained data supporting the main findings.

**Table II.** The Encounter between the General Practitioner and the patient and the different steps of the analysis.

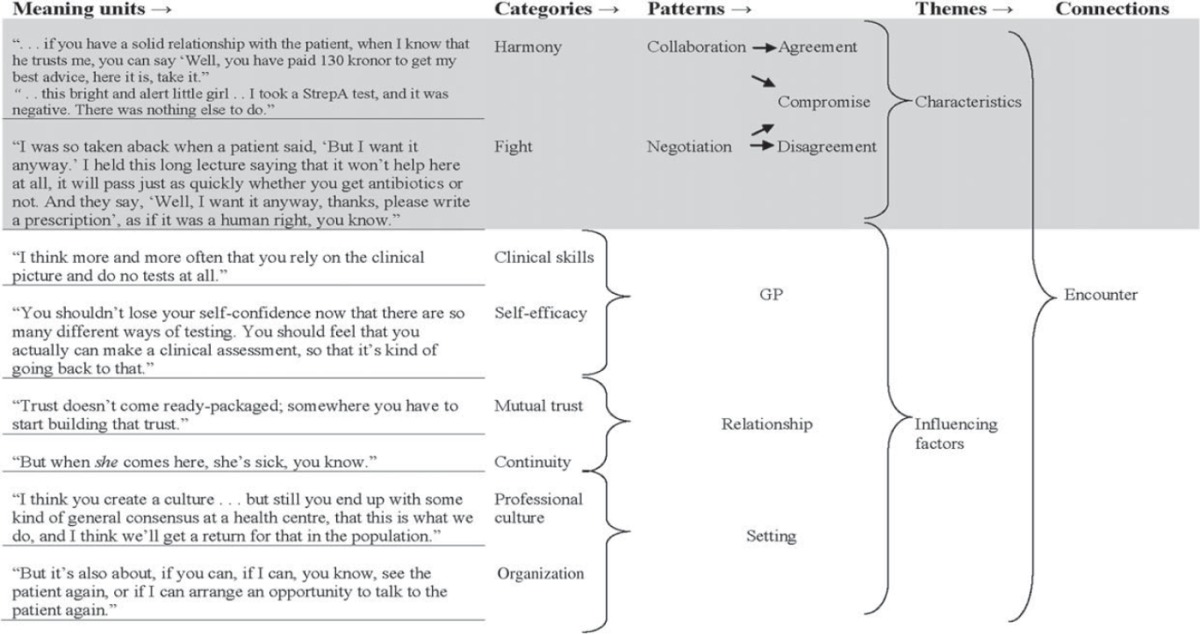

When referring in the results to the participants’ statements, these are written in the past tense, while interpretations of statements are in the present tense.

### Ethical considerations

According to Swedish legislation, ethical approval was not necessary for this study since it was considered a quality improvement project. All participants were informed that participation was voluntary, that they could withdraw at any time, that all data would be handled confidentially, and that the results would be presented in a non-identifiable way.

## Results

**Figure 1.**
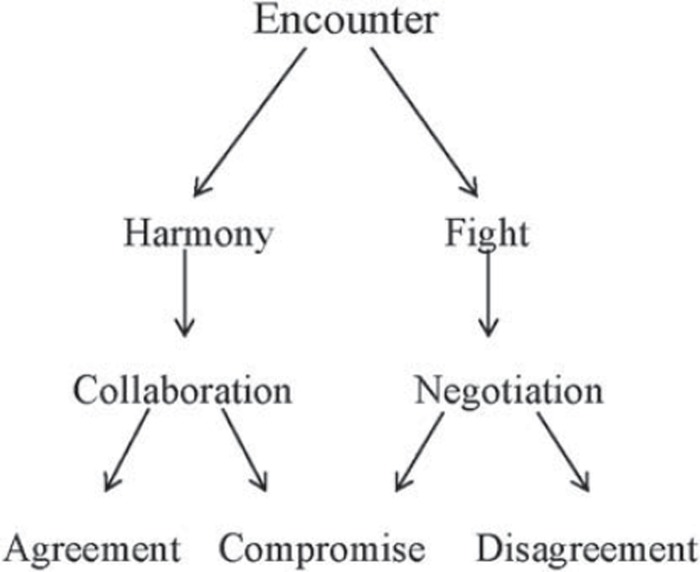
Characteristics of the encounter between the GP and the patient.

**Figure 2.**
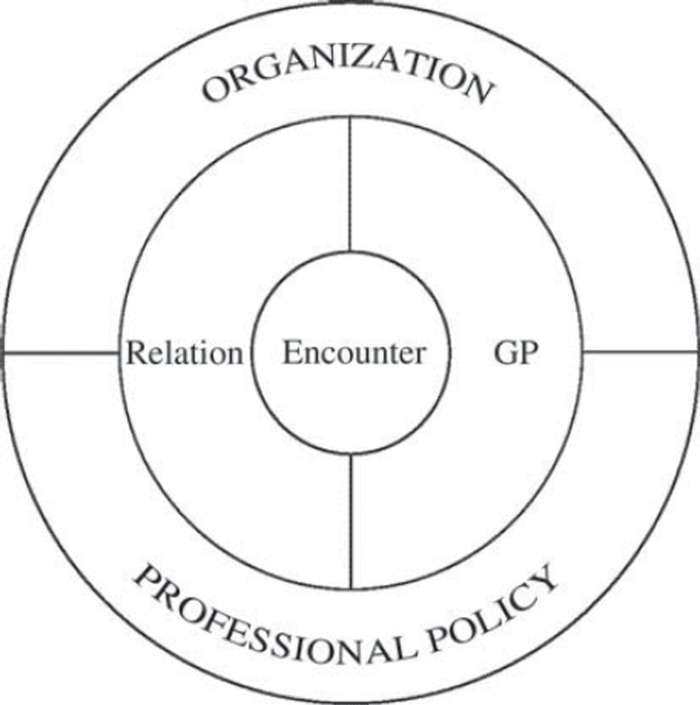
Factors influencing the encounter between the GP and the patient.

We found that the most significant and important factor concerning antibiotic prescribing for RTIs is what happens when the GP meets the patient (Table II). Our main finding concerns two dimensions of this encounter: *characteristics* of the meeting depending on the interaction with the patient (Figure 1) and *influencing factors* (Figure 2).

### Characteristics of the encounter

When the expectations of the GP and the patient are congruent, the encounter is recognized as smooth and in *harmony* with *collaboration* until *agreement* or *compromise* is reached. When the expectations do not match, it is characterized as a *fight* where *negotiation* is needed. Sometimes negotiation leads to a *compromise*, sometimes it ends in *disagreement*.

### Factors influencing the encounter

When looking closer into the encounter between the GP and the patient and factors influencing the meeting, we found that the participants talked about three main factors: the GPs themselves, the relationship with the patient, and the setting in which the encounter takes place, each with two to six sub-factors (Table III).

**Table III.** Factors and sub-factors influencing the encounter between the GP and the patient.

Factors	Sub-factors
GP factors	Clinical skills Knowledge of guidelines Self-efficacy Autonomy Authority Strategies
Relationship factors	Mutual trust Learning Continuity Flexibility
Setting factors	Professional culture Organization of work

*GPs themselves.* Our interpretation is that *clinical skills* and *knowledge of guidelines* are important factors for making decisions about whether to prescribe antibiotics for RTIs or not. *Self-efficacy*, i.e. not losing confidence in one's own capability, is important in spite of all possible tests, near-patient tests and others. Such clinical tests are not always used. Professional *autonomy* was expressed to be of importance for prudent prescribing of antibiotics, as was the GP's professional *authority* in the clinical situation for trustworthiness with the patient. Prudent prescribing is not always about reducing prescribing rates, but prescribing for the right indications. All GPs have their own *strategies* for making the consultation a success. Our participants talked about *short cuts* – being lazy and obedient to guidelines for treating RTIs, and guidelines and *rules of thumb* could save time and energy. It was considered useful to give the patient a couple of minutes’ extra *time* for reasoning about antibiotics and resistance problems. *Safety netting* through possible follow-up was mentioned as a strategy in itself, but difficult when meeting the patient in the emergency centre. *Information about the risks* of excessive use of antimicrobial drugs was considered a useful strategy.

*Relationship with the patient*. The participants expressed views on the relationship between the GP and the patient. Our interpretation is that continuity of care and mutual trust between the GP and the patient are also very important factors in the matter of antibiotic prescribing in general and for RTIs in particular. *Continuity* promotes diagnostic accuracy through personal knowledge, and a time-consuming explanation could be a wise investment. In contrast, anonymous and temporary physician staff, who only stay for a short while, are considered not trustworthy. Unnecessary antibiotic prescribing creates future demands. The patient becomes “mislearned”, i.e. *learning* the wrong behaviour and expectations. *Mutual trust* is necessary for the patient to understand when he/she is advised to “wait and see” and for the GP to advocate a delayed prescription.

The participants said that to fit the individual, the care provided must be characterized by *flexibility*. Rigid adherence to guidelines was not regarded as a correct interpretation of evidence-based medicine, as a GP always has to take into account the patient in front of him/her. Professional performance is about being able to judge what is best for the patient.

*Setting of the* encounter. The *organization* of the work may facilitate or complicate the rational use of antibiotics for RTIs.

A facilitating factor that was mentioned is the overall consensus at the primary health care centre (PHC) about how to treat patients with RTIs. Overall consensus and a common *professional culture* are achieved through local professional discussions and exchange of experience. Teamwork with primary nurse assessment and return visits for follow-up are also considered facilitating.

The availability of ordinary GPs is a facilitating factor as well. A primary care practice with ordinary GPs promotes *continuity* of care. Many Swedish physicians today are hired for a shorter period of time without a list of patients of their own and they are, according to our participants, the ones who usually meet the RTI patients, a kind of *fragmentation* of the workload. According to the participants, they seem to have a tendency to prescribe more antibiotics for RTIs than the ordinary GP.

GPs who reported that they normally follow the guidelines admitted that they prescribe more antibiotics when on call or at the emergency centre. This is due to lack of time and possibility to follow up but also to the absence of personal knowledge of the patient: “I'm Dr Jekyll *and* Mr Hyde.”

## Discussion

### Summary of main results

The decision to prescribe antibiotics or not takes place in the encounter between the GP and the patient. This is initially characterised by harmony or fight and the ensuing process by collaboration or negotiation, resulting in agreement, compromise, or disagreement. Several factors influence the meeting and contribute to enhancing the conditions for rational prescribing. These conditions are connected to the GP, the relationship with the patient, and the setting; the latter comprises the organization as well as the professional culture. The findings indicate that there are synergies between the factors.

### Strengths and limitations

This study is part of a multi-method approach to deepen the understanding of circumstances favourable to rational antibiotic prescribing. Focus groups were chosen for data collection. We planned for and conducted two groups and were prepared for a third if the data achieved proved to be insufficient. This method has the advantage of producing ample data in a limited time, and the group process between the participants stimulates the sharing of experiences. The participants were strategically chosen among those who participated in both registrations in the Happy Audit project, indicating that they were actively and practically engaged in rational prescribing and thus could be expected to prescribe antibiotics more prudently. We recruited participants to get as broad a representation as possible from the Happy Audit material, which means that the invited participants represented both urban and rural areas as well as low-prescribing and high-prescribing districts/areas. According to the literature there is no magic number concerning sample size in focus-group interviews. A common recommendation is two to five groups [22]. Sample size is not, according to Crabtree and Miller [21], “the determinant of research significance in a qualitative study; the major concern is with information richness”.

The GPs had voluntarily participated in the Happy Audit project including the intervention. It is therefore likely that they have more knowledge and are more reflective concerning antibiotics than the average practitioner. In spite of this, they share numerous examples of acting contrary to the guidelines. Thus, we believe that these experiences are transferable at least in a Swedish context.

The data reflect the views and experiences of the participating GPs. The expectations and experiences of the patients are illustrated only indirectly. The findings cannot be generalized, but this is never the intention in qualitative studies, the main aim of which is to contribute to increased understanding. It is nevertheless reasonable to assume that our findings can be transferred to similar contexts in general practice.

### Comparison with existing literature

The findings of this study indicate that beliefs concerning the demands and expectations of patients influence the prescribing pattern. This is corroborated by similar results in previous studies [5,23].

One result was the importance of the knowledge level of the GP, also corroborated in a previous study [24]. The findings indicate that continuity is favourable for the relationship between GP and patient, but it is obvious from another study [25] that when patients do not get well, continuity is not sufficient.

The participants in this study revealed experiences concerning the consequences of the organization for prescribing of antibiotics. The organization of primary care may be important in at least two ways. It can facilitate a good relationship between the GP and the patient by promoting continuity. A stable relationship based on continuity increases the chances of a smooth encounter. Thus, inappropriate organization may indirectly contribute to increased antibiotic resistance.

Second, stable staff at a PHC is a prerequisite for the formation of a professional culture with a local common policy. This can be labelled continuity on the aggregated level. The findings of this study indicate that a common policy in a primary care centre is a success factor in the task of improving prescribing habits. Corresponding results have been found in previous research as well as in a focus-group study on similar topics among Happy Audit participants in Lithuania and the Kaliningrad region [26,27].

A professional culture with a common policy may not simply be regarded as a result of adherence to guidelines, but also as an autonomous attitude: a genuine desire to do things right and make a difference [28].

An alternative path to achieving a common policy might be to have professional meeting places outside the PHC, for instance continuing medical education (CME) groups. We believe that this also may improve the autonomous attitudes of GPs. A common professional policy combined with skill and knowledge of guidelines entails synergies between the bottom-up and top-down approaches. If this process includes both GPs and nurses, a feasible way to optimal antibiotic prescribing for RTIs is created.

In several countries pay for performance is used as a tool to improve quality in practice, for instance regarding antibiotic prescribing. The results are ambiguous, and there is widespread debate about whether financial incentives are effective for this purpose [29,30].

In future research we intend to deepen the understanding of a professional culture and continuity. We also plan to study the effects of the freedom-of-choice reforms in Sweden and the increase in drop-in clinics in the PHCs.

Our findings emphasize the importance of the encounter between the GP and the patient for decisions concerning antibiotic prescribing. A stable relationship between the two and the GP's knowledge of guidelines contribute to prudent prescribing. Furthermore, the importance of appropriate organization of primary care, which promotes continuity of care and encourages professional autonomy, is demonstrated.

### Authors’ contributions

ELS and AB were equally involved in study design, conducted and analysed the focus groups and interviews, wrote the paper, and will serve as guarantors for the integrity of the data. CH and KH did the literature search. MT contributed to editing the paper. All authors have read and approved the final manuscript.
